# Recent developments in *Salvia miltiorrhiza* polysaccharides: Isolation, purification, structural characteristics and biological activities

**DOI:** 10.3389/fphar.2023.1139201

**Published:** 2023-03-03

**Authors:** Lei Luo, Juan Xue, Zheng Shao, Zhang Zhou, Wenqian Tang, Jinxin Liu, Hongfei Hu, Fan Yang

**Affiliations:** ^1^ Department of Health Management Center, Hubei Provincial Hospital of Traditional Chinese Medicine, Wuhan, China; ^2^ Department of Gastroenterology, Hubei Provincial Hospital of Traditional Chinese and Western Medicine, Wuhan, China; ^3^ School of Clinical Medical, Hubei University of Chinese Medicine, Wuhan, China; ^4^ Department of Anesthesiology, Wuhan Fourth Hospital, Wuhan, China

**Keywords:** Salvia miltiorrhiza, polysaccharides, extraction methods, purification, structural characteristics, bioactivities

## Abstract

In recent years, natural polysaccharides have attracted more and more attention and research because of their value in the medicine, beauty and food fields. *Salvia miltiorrhiza* is a traditional Chinese herb that has been used for thousands of years and has antidiabetic, antifibrotic, neuroprotective, antioxidation, anti-inflammatory and other effects. It mainly includes rosmarinic acid, tanshinone I, tanshinone IIA, tanshinone IIB, procatechualdehyde, polysaccharide and salvianolic acids. *Salvia miltiorrhiza* polysaccharide is a polysaccharide extracted and isolated from *Salvia miltiorrhiza* and has diverse biological functions, including antioxidation, anti-tumor, hepatoprotective, anti-inflammatory, immune regulatory and cardioprotective effect. In this review, the extraction, purification, structural characterization and biological activity of SMPs are summarized and new perspectives for the future work of SMPs were also proposed, we hope our research can provide a reference for further research on SMPs.

## 1 Introduction

**FIGURE 1:**
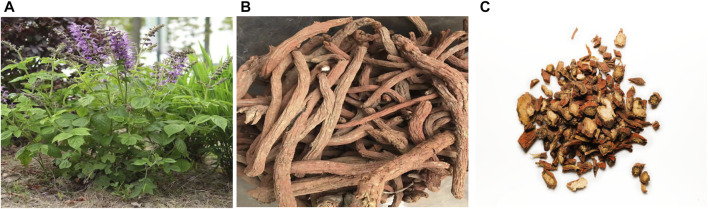
**(A)** The above-ground portion of *salvia miltiorrhiza*; **(B)** The rhizome of *salvia miltiorrhiza*; **(C)** commercial herbal pieces of *salvia miltiorrhiza*.

*Salvia miltiorrhiza* (Figure 1), also known as blood ginseng, red ginseng and red roots, is a dicotyledonous erect perennial herb of the genus Salvia in the Labiaceae family and is mainly distributed in Anhui, Shanxi, Hebei and Sichuan and other provinces of China and also in Japan. The dried root of *Salvia miltiorrhiza*, also known as *Salvia miltiorrhiza*, is a famous traditional medicinal plant in China and was first included in Shennong’s Classic of Materia Medica (Shennong Bencao Jing) (Su et al., 2016). Traditional Chinese medicine holds that *Salvia miltiorrhiza* is mildly cold and bitter, and the main efficacy of *Salvia miltiorrhiza* is to promote blood circulation and regulate menstruation, remove blood stasis and relieve pain, cool blood and carbuncle and remove irritability and calm the mind (Qiao et al., 2011). It is widely used in the treatment of irregular menstruation, dysmenorrhea, insomnia, chest pain, injury and other diseases (Ma et al., 2015; Zhang Z. et al., 2019). With more and more chemical and pharmacological researches on *Salvia miltiorrhiza*, researchers have found that *Salvia miltiorrhiza* exhibit diverse biological functions, such as anti-tumor, anti-oxidative stress, anti-thrombosis, anti-inflammatory, anti-liver fibrosis and anti-diabetes activities (Guo et al., 2014; Zhou et al., 2014; Jiang et al., 2019; Petitjean et al., 2022). These effects are attributed to its wide range of bioactive ingredients, including tanshinone I, tanshinone IIA, tanshinone, procatechualdehyde, polysaccharide, salvianolic acids and other chemical substances (Jia et al., 2019; Kapalavavi et al., 2021; Leng et al., 2022).

Polysaccharides are important components present in organisms such as animals, plants and microorganisms and are biological macromolecules made up of not less than 10 monosaccharides connected in the sequence of glycosidic bonds (Xie et al., 2016; Chen and Huang, 2018). Recently, plant polysaccharides have become a hot spot for research due to their unique properties, including high biological activity and low toxicity (Shao et al., 2020). *Salvia miltiorrhiza* polysaccharide (SMP) is the main component and important active component of *Salvia miltiorrhiza*, which has carious pharmacological activities, such as hepatic protection, antioxidant, anti-tumor, immune regulation and cardioprotection (Song et al., 2008; Song et al., 2013; Zhu et al., 2020). However, different raw materials and extraction and purification methods are used to obtain SMPs with various structural and pharmacological activities.

To our knowledge, no review concerning SMPs is available. Therefore, this paper comprehensively reviewed the recent advances in the isolation, purification, structural characteristics and pharmacological activities of SMPs in recent years and looks forward to its future research direction, which will provide a insight for in-depth understanding, development and utilization of SMPs.

## 2 Extraction and purification methods of SMP

**FIGURE 2:**
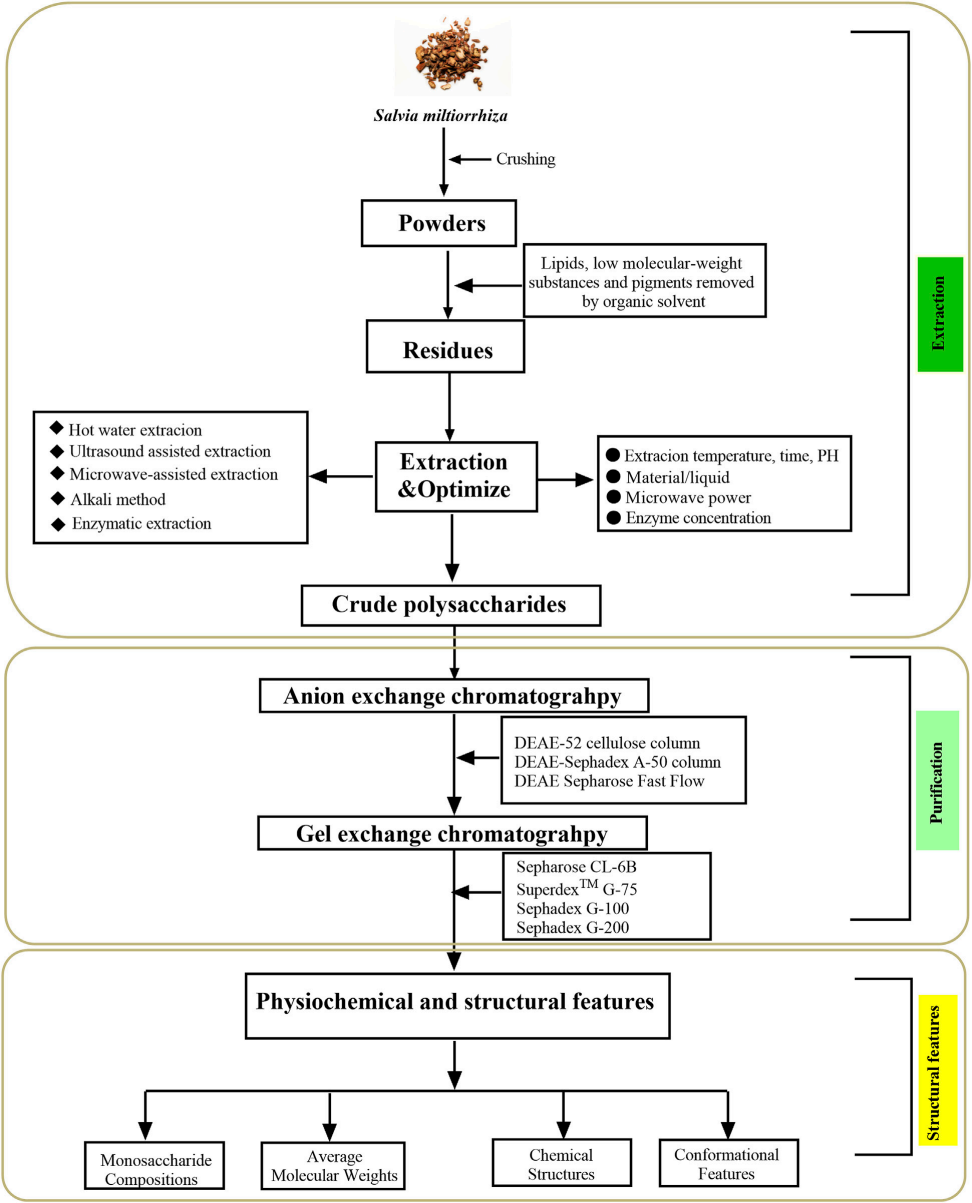
The flowchart of the extraction, purification, and structural features of *Salvia miltiorrhiza* polysaccharides.

Polysaccharides are substances with high polarity that are water-soluble and insoluble in ethanol (Li et al., 2021). Generally speaking, the chemical properties of the extracted components and the influence of impurities should be considered in the selection of extraction and purification methods (Xu et al., 2022). Therefore, selecting appropriate extraction and purification methods of polysaccharides could improve the extraction rate of polysaccharides more efficiently without destroying the structure of polysaccharides. In recent years, with the continuous development of research methods, the extraction and purification efficiency of SMPs has been improved. Figure 2 schematically illustrate the extraction, purification, as well as structural features of SMPs.

### 2.1 Extraction of SMP crude polysaccharide

Considering that SMP is a water-soluble polysaccharide, the hot water extraction method is an effective and classical method for the separation of SMP. Ethanol precipitated polysaccharides are suitable for almost all water-soluble polysaccharides, including SMP. In brief, dried *Salvia miltiorrhiza* roots were refluxed in a Soxhlet extractor with 95% ethanol to remove lipophilic molecules and pigments, followed by a 20-fold volume of hot distilled water extracted three times at 100°C for 2 h each. After filtering through gauze, 4 times the volume of 95% ethanol solution was added to mix, and the precipitate was placed in a refrigerator at 4°C for 12 h to obtain SMP crude polysaccharide (Wang et al., 2014). In another study, the SMP was extracted with distilled water at 80°C and precipitated with 75% ethanol overnight at 50°C (Song et al., 2013). Some scholars removed fat pigment and low molecular weight matter from the root of *Salvia miltiorrhiza* through 95% ethanol, and then SMP was obtained by water extraction and alcohol precipitation, and the yield was 1.8% (Chen et al., 2017). On the basis of Box-Behnken central combination design and response surface methodology (RSM), deionized water was used to extract SMP. The results showed that when the ratio of material to liquid was 32 mL/g, the extraction time was 2.6 h and the extraction temperature was 89°C, the yield of SMP was 27.32% ± 0.4% (Jiang et al., 2015). Hot water extraction is a commonly used extraction method, which is economical, environmentally friendly and operable. However, high temperature and long time extraction will lead to the destruction of polysaccharide structure, reduce its pharmacological activity, and reduce the extraction rate (Hromádková et al., 2002). In order to overcome these shortcomings, different techniques were studied, including ultrasonic extraction, microwave-assisted extraction and enzyme extraction.

The ultrasonic extraction method uses an ultrasonic method to extract polysaccharides, which has the advantages of no high temperature, high extraction efficiency, good safety and simple operation (Afshari et al., 2015; Luft et al., 2021). Jiang et al. (2014) used the combination of the response surface method and ultrasonic method to extract SMP and found that the extraction rate of SMP is the highest when the ultrasonic power is 180 W and the extraction temperature is 54°C. When the liquid‒solid ratio was 22 mL/g, the extraction temperature was 60°C, the ultrasonic power was 205 W, and the ultrasonic time was 125 min, the SMP yield reached 5.99% ± 0.09% (Ji et al., 2021). Because of the mechanical shear effect of ultrasonic extraction, the structure of SMP may be damaged if ultrasonic extraction time is too long. In addition, due to the limitation of instruments, ultrasonic extraction can not be widely used in industrial field (Zheng et al., 2021).

Microwave-assisted extraction destroys cell wall by microwave heating and pressure, thus promoting the release of intracellular polysaccharides. Compared with traditional methods, microwave-assisted extraction has a higher yield and lower energy consumption (Zeng et al., 2012). RSM based on Box-Behnken optimizes the extraction of SMP from microwave power, extraction time, solvent-to-solid ratio and the concentration of ethanol. The results showed that the microwave power was 1200 W, the extraction time was 12 min, the solvent solid ratio was 38 mL/g, the ethanol concentration was 86%, and the SMP extraction rate was the highest (Meng et al., 2022).

Enzymatic extraction is a method of extracting polysaccharides from plant cell walls by enzymatic hydrolysis, which has the advantages of friendly environment, low energy consumption, high extraction efficiency and high extraction rate (Wijesinghe and Jeon, 2012). An SMP of 2.51 mg/g was obtained by 0.5% cellulase extraction at 65°C for 120 min (Yang and Zhang, 2016). Wu et al. (2012) extracted four kinds of SMPs (SMP1, SMP2, SMP3 and SMP4) by the hot water method, ultrasonic method, alkali method and enzymatic method. It was found that the physical and chemical properties of the four polysaccharides were basically similar but had different three-dimensional structures. The process of enzyme extraction is mild and has no damage to the molecular structure of polysaccharide, but the stability of enzyme is affected by temperature, PH value, reaction time and so on (Chen et al., 2013). The extraction methods of SMPs is shown in Table 1.

**TABLE 1:** A summary of the extraction methods of SMPs.

Compound name	Extraction methods	Temperature	Solid-liquid ratio	Time (min)	Power	Content	References
SMWP-1	Hot water method	89°C	32 mL/g	156	—	27.32% ± 0.4%	Jiang et al. (2015)
SMP-U1	Ultrasonic method	54°C	—	32	180w	40.54% ± 0.25%	Jiang et al. (2014)
cSMEP	Ultrasonic method	60°C	22 mL/g	125	205w	5.99% ± 0.09%	Ji et al. (2021)
SMP-1	microwave-assisted extraction	—	38 mL/g	12	1200w	14.11%	Meng et al. (2022)
SMP	Enzyme method	65°C	—	120	—	2.59 mg/g	Yang and Zhang (2016)
SMP-1	Hot water method	70°C	1:20	—	—	—	Wu et al. (2012)
SMP-2	Ultrasonic method	Room temperature	1:20	40	—	—	Wu et al. (2012)
SMP-3	Alkali method	Room temperature	1:20	—	—	—	Wu et al. (2012)
SMP-4	Enzyme method	60°C	1:10	120	—	—	Wu et al. (2012)

### 2.2 Purification of SMP

The *Salvia miltiorrhiza* was initially extracted to obtain the SMP crude polysaccharide contains proteins, pigments and other impurities, which need to be further removed to obtain purified SMP. The purification methods, such as deproteinization, decolorization, gel filtration and ion exchange chromatography, were used to further purify and obtain higher quality refined polysaccharide (Zeng et al., 2019; Fang et al., 2020). The salvage method is the primary method for deproteinization. Activated carbon and hydrogen peroxide decolorization are commonly used decolorization methods at present. Column chromatography methods commonly used in the purfication of polysaccharide include anion exchange chromatography, cation exchange chromatography, and gel filtration chromatography (Zhang et al., 2021).

The SMP crude polysaccharide extracted with deionized water was purified by a diethylaminoethyl (DEAE)-Sepharose CL-6B column. After elution, dialysis and lyophilization with 0.5 mol/L NaCl solution and purified on Sephadex G-100 gel permeation column to get uniform SMP (Jiang et al., 2015). The SMP extracted by the microwave method was deproteinized by the Sevage method and further purified by a DEAE Sepharose Fast Flow (2.6 × 15 cm) column and Sephadex G-100 chromatography to obtain SMP with an average molecular weight of 6,087 Da (Meng et al., 2022). The protein was removed by the salvage method combined with the papaya protease method, and after ethanol precipitation, it was separated by the DEAE cellulose anion exchange method, concentrated and purified on a Sephadex G-100 column to obtain 8.26% SMP (Liu et al., 2013).

## 3 Physiochemical and structural features of SMP

Natural polysaccharides are high molecular weight polymers formed by glycosidic bonds (Ain et al., 2022). The structural characteristics of polysaccharides are understood by measuring their monosaccharide composition, molecular weight, conformation of glycosidic bonds and the position and sequence of carbohydrate linkage. It is of great importance to understand their pharmacological activities and structure-activity relationships. The techniques for identifying the structure of polysaccharides mainly include infrared spectroscopy (IR), high-performance gel permeation chromatography (HPGPC), high-performance liquid chromatography (HPLC), nuclear magnetic resonance (NMR), gas chromatography (GC) and gas chromatography‒mass spectrometry (GC‒MS). The monosaccharide compositions, molecular weights, Possible Structures and biological activities were exhibited in Table 2.

**TABLE 2:** Monosaccharide compositions, molecular weight, possible structures and biological activities of polysaccharides from *Salvia miltiorrhiza.*

NO.	Compound name	Monosaccharide compositions	Molecular weight (kDa)	Possible structures	Bioactive activities	References
1	SMP1	Gal, Glu, Fuc, Rha, Ara and Man in a molar ratio of 1.0: 1.2: 0.3: 1.5: 1.3: 1.9	550	ND	Cardioprotective, antioxidant and Anti-apoptosis	Song et al. (2013)
2	SMPA	Gal, Glc, Rha, Man, and GalUA in a molar ratio of 2.14: 1.42: 1.16: 2.15: 1	43	ND	Anti-tumor and immunomodulatory activity	Wang et al. (2014)
3	SMP	Ara, Gal, Glu, Rham, and GalUA in a molar ratio of 4.79: 8.24: 3.26: 1: 6.52	ND	ND	Anti-tumor and immunomodulatory activity	Chen et al. (2017a)
4	SMWP-1	Glu, Xyl, Man, and Galin a molar ratio of 0.34:0.28:0.27:0.11	527	typical characteristics of α-dominant configuration	antioxidant	Jiang et al. (2015)
5	SMP-U1	Man, Rib, Xyl, Ara, Glu, and Gal in a molar ratio of 1.95: 0.22: 0.10: 1.57: 1.45: 1.34	569	β- and α-configuration	Antioxidant, antiproliferation and anti-tumor	Jiang et al. (2014)
6	SMEPs	Gal, Glu, Fru in a molar ratio of 1.00:0.45:0.28	6.5	α/β-type glycosidic bonds	immunomodulatory	Ji et al. (2021)
7	SMP1	Glu, Gal, and Fru in a molar ratio of 1:1.67:1.12	6.087	structural fragment: β-D-fruf, α-D-galp, α-D-galp, α-D-glcp	Antioxidant	Meng et al. (2022)
8	SMP-W1	Man, Rha, Ara, Glc and Gal in a molar ratio of 2.14: 2.35: 1.27: 0.99: 1.11	690	ND	Anti-tumor and immunomodulatory activity	Liu et al. (2013)
9	PSMP-2	Rha, GalA, Gal, and Ara in a molar ratio of 6.15: 55.98: 21.27: 16.69	1280	contained five major glycosidic bonds, including (1→)-linked-Ara, (1→2, 4)-linked-Rha, (1→4)-linked-Gal, (1→6)-linked-Gal, (1→3, 6)-linked-Gal	Antioxidant, Anti-apoptosis and Cardioprotective	Jing et al. (2022)
10	SMP	Gal, Glc and GalUA in a mole percentages of 64.5%, 31.1% and 4.4%	120	ND	Anti-tumor and antioxidant	Wang et al. (2018)
11	SMRR	GalA, Ara, Gal, Rha and Glc ina molar ratios of 17.9: 1.3: 1.7: 1.2: 1	32.6	ND	improving intestinal function and hepatoprotective	Li et al. (2022)
12	PSF-W-1	Man, Glu and Gal in molar ratios of 1.00: 4.86: 2.25	36.13	backbone of →4)-β-D-Glcp-(1→6)-α-D-Galp-(1→4)-β-D-Manp-(1→6)-α-D-Galp-(1→ with two side chains β-D-Glcp-(1→4)-β-D-Glcp-(1→ attached to O3 of 1,6-α-D-Galp	Stimulants for the synthesis of tanshinone	Wu et al. (2019)
13	SMP	Gal, Glu, Ara, Rha, Man and Fuc in a mole percentages of33.14%, 17%, 1.05%, 0.36%, 0.18% and 0.05%	2.8	ND	antioxidant	Chen et al. (2022)
14	SMWP-U&E	Ara, Fru, Man, Glc and Gal in a mole percentages of 3.72%, 4.11%, 6.18%, 32.08% and 53.91%	507	structural fragment:β-D-Fruf, β-D-Glcp, α-D-Galp, β-L-Xylp, α-D-manp, α-D-Glcp	improving intestinal function, antioxidant and immunomodulatory activity	Jiang et al. (2020)

ND: Not detected. Abbreviations: Glc, glucose; Gal, galactose; Man, mannose; Ara, arabinose; Xyl, xylose; Rha, rhamnose; Glu, glucose; Fru, fructose; Rib, ribose; (GalA, GalUA), galacturonic acid; Fuc, fucose.

### 3.1 Monosaccharide compositions

The monosaccharide composition of polysaccharides is usually determined and quantified by complete acid hydrolysis, derivatization, GC and HPLC (Zhang X. et al., 2019). Previous studies have shown that most polysaccharides are made up of galactose, mannose, glucose, rhamnose, xylose and arabinose in different molar ratios. Different sources of *Salvia miltiorrhiza*, different extraction and purification methods, and different detection methods lead to different monosaccharide compositions in SMPs.

SMP is a heterosaccharide composed of mannose, galactose, arabinose, glucose, xylose and other monosaccharides. The analysis methods of monosaccharide composition commonly by GC-MS, GC and HPLC. Jiang et al. (2015) isolated the polysaccharide SMWP-1, and GC‒MS analysis showed that it was composed of glucose, xylose, mannose and galactose, with a molar ratio of 0.34: 0.28: 0.27: 0.11. A purified polysaccharide, PSMP-2, was obtained by hot water extraction, ethanol precipitation and further purification on a Sephadex G100 column. Jing et al. (2022) isolated the polysaccharide PSMP-2, whose monosaccharide composition mainly consist of rhamnose, galacturonic, galactose, and arabinose in molar percentages of 6.15: 55.98: 21.27: 16.69. Through GC analysis, Geng et al. (Geng et al., 2015a). found that *Salvia miltiorrhizae* polysaccharide SMP1 was composed of galactose, glucose, caramel, rhamnose, arabinose and mannose, with a molar ratio of 1.0: 1.2: 0.3: 1.5: 1.3: 1.9. SMP is an acidic heterosaccharide consisting of D-galactose, D-glucose and D-galacturonic acid with a molar ratio of 15.03: 7.14: 1.00 (Wang et al., 2018).

### 3.2 Average molecular weights

Different techniques, including HPLC and HPGPC, were widely applied to determine the average molecular weights of polysaccharides (Wang X. Y. et al., 2019; Zhao et al., 2019). Under different experimental conditions, the molecular weights of SMPs ranging within 2–1300 kDa, which is summarized in Table 2. SMP-W1 was obtained from the crude polysaccharide solution of *Salvia miltiorrhiza* by deproteinization, further separation and purification on a DEAE cellulose column and a Sephadex G-100 chromatographic column, and its molecular weight was 6.9 × 10^2^ kDa, as determined by HPGPC (Liu et al., 2013). Studies have proven that SMP1 was a homogeneous polysaccharide, and its average molecular weight was determined to be 6.087 kDa by HPGPC. Li et al. (2022) obtained SMP crude polysaccharide SMRR by water extraction and alcohol precipitation and further obtained SMRR by gel filtration on a DEAE-Sepharose Fast Flow anion exchange separator. According to the gel permeation chromatography (GPC), the average molecular weight of SMRR was 32.6 kDa. Another study found that the average molecular weight of SMWP-1 was 5.27 × 10^2^ kDa (Jiang et al., 2015).

### 3.3 Chemical structures

**FIGURE 3:**
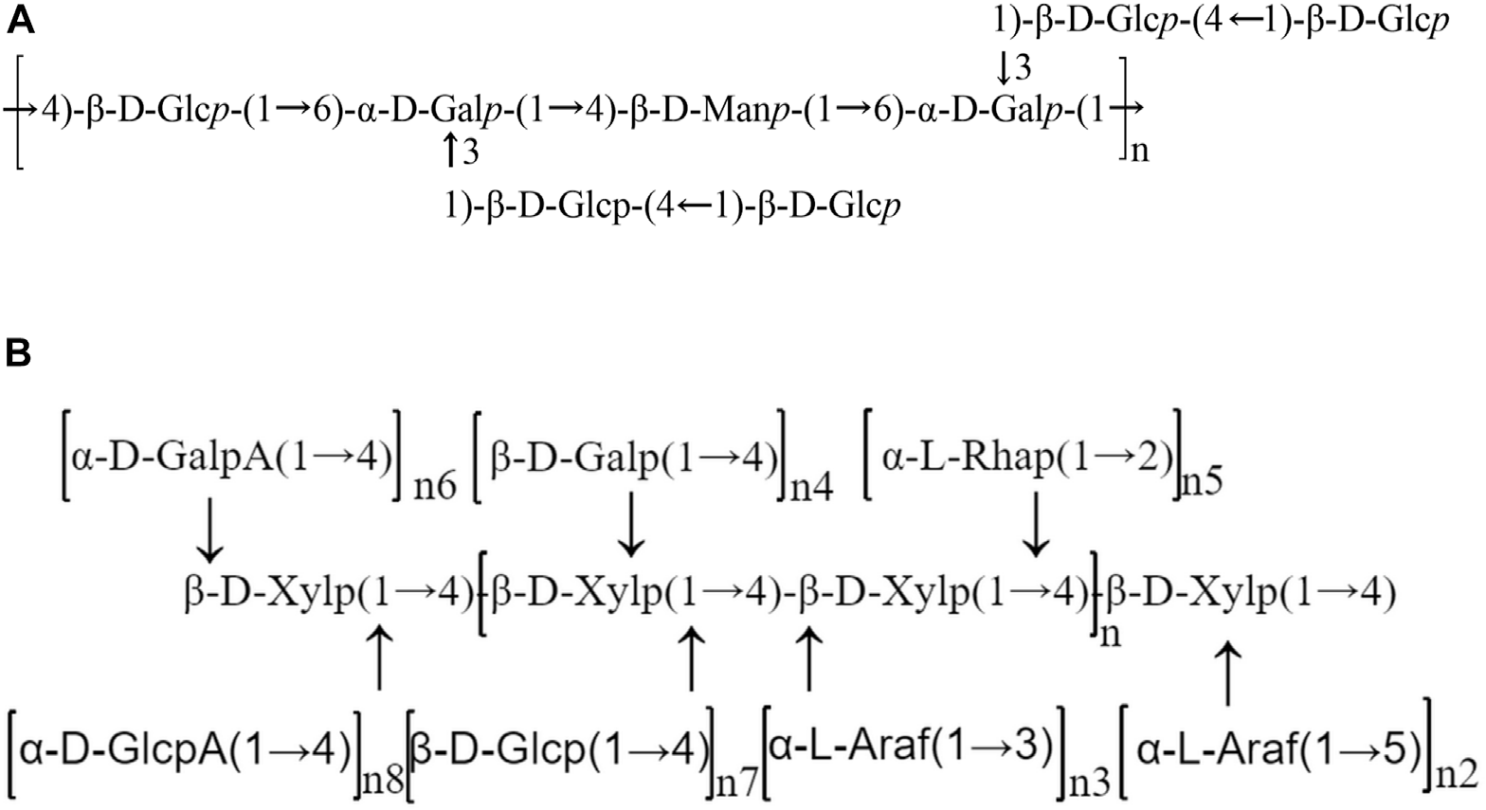
Schematic structures of *Salvia miltiorrhiza* polysaccharides. **(A)** Chemical structure of PSFP-W-1 (Wu et al. (2019)); **(B)** The possible structure model of HBPs (Zhao et al. (2020)).

Apart from their monosaccharide components and molecular weight, the structural feature of polysaccharides is also of great significance for understanding their pharmacological activities. The chemical structure of SMPs have rarely been reported in the literature. The chemical structure of SMPs was studied by GC‒MS, methylation analysis, Fourier transform infrared (FTIR) spectroscopy and NMR analysis. The possible backbone and branches of SMPs are summarized in detail in Table 2 and Figure 3.

Through methylation analysis, infrared spectroscopy and NMR analysis, it was determined that the skeleton of neutral *Salvia miltiorrhiza* heteropolysaccharide PSF-W-1 was → 4)- β- D-Glcp-(1→6)- α-dgalp -(1→4)- β-d - manp -(1→6)- α- D - galp - (1 →) with two side chains β- D-Glcp-(1→4)- β- D-Glcp - (1 → to O3 of 1,6- α- D—Galp (Wu et al., 2019). Jing et al. (2022) found that a polysaccharide from *Salvia miltiorrhiza,* named PSMP-2, has five main glycosidic bonds through methylation analysis and GC analysis, including (1 →)—linked Ara, (1 → 2, 4)—linked Rha, (1 → 4)—linked Gal, (1 → 6)—linked Gal, (1 → 3, 6)—linked Gal, with a molar ratio of 5.98: 1.45: 72.23: 16.40: 3.94. Zhao et al. (2020) used ^1^H/^13^C NMR analysis of the structure of hemicellulose-based polysaccharides isolated from *Salvia miltiorrhiza* named HBPs, and the backbone structure indicated that 4-β-D-Xylp served as the main chain connected by the 3-α-L-Araf or 5-α-L-Araf-1, 4-β-D-Galp, and β-D-Glcp branches, as well as the α-L-Rhap, α-D-GalpA and α-D-GlcpA fragments. Methylation linkage analysis and GC‒MS results showed that the backbone chains of SMP1 are mainly (1→3,6)-β-D-mannopyranosyl (Residue-A), (1→6)-β-D-glucopyranosyl (Residue-B), and (1→3,6)-β-D-galactopyranosyl (Residue-C) residues (Geng et al., 2015a).

### 3.4 Conformational features

The secondary structure refers to the three-dimensional structure formed by the penetration bond generated by the molecular structure of the polymer and the physical force penetrating the space, and “conformation” is used to define this property (Ain et al., 2022). The conformation of biopolymers can be spherical, rod, single helix, double helix, coil (Eder et al., 2021). Understanding the conformational characteristics of polysaccharides is very important for understanding their biological activities. The conformational characteristics of polysaccharides are usually analyzed by atomic force microscope (AFM) and scanning electron microscope (SEM) (Fan et al., 2020; Gong et al., 2022).

Jiang et al. (2015) analysed the surface morphology of SMWP-1 by SEM and showed that its structure was loose and honeycomb, and the surface was composed of single spherical particles with a regular distribution. The SEM image of SMP1 obtained by microwave-assisted extraction shows a spongy structure with a rough surface (Meng et al., 2022). The SMPs obtained by different extraction methods from the same source have different characteristics. SEM images revealed that SMP-1 extracted by the hot water method had a flat surface, SMP-2 extracted by the ultrasonic method was rough and had obvious large wrinkles, while SMP-3 extracted by the alkali method was sponge-like and the surface of SMP-4 extracted by the enzymatic method was very smooth and had distinct pore openings 2–5 μm in diameter. (Wu et al., 2012).

## 4 Biological activity

**FIGURE 4:**
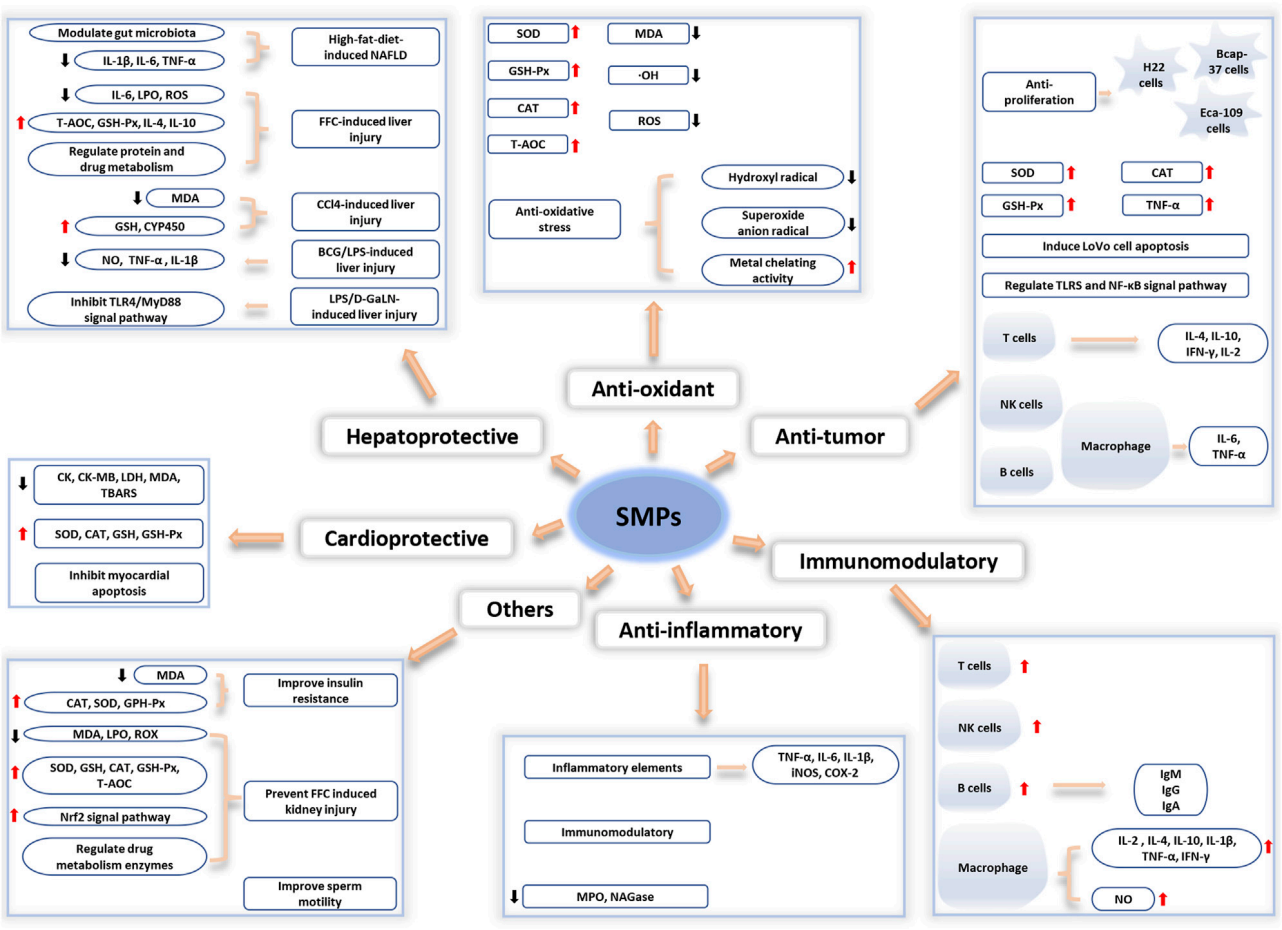
The biological activities and molecular mechanisms of SMPs.

Studies have shown that polysaccharides are an important biologically active ingredient extracted from *Salvia miltiorrhiza*, which has beneficial effects on human health. The complex diversity of polysaccharide structures results in various biological activities. The various biological actions of SMPs, including anti-tumor, hepatoprotection, antioxidant, immunomodulatory, anti-inflammatory activities and other biological activities. The biological activities and molecular mechanisms of SMPs are shown in Figure 4.

### 4.1 Antioxidant effect

Scientific studies have shown that excessive oxygen free radicals in the body are related to the occurrence and development of many diseases, such as tumor, aging and diabetes, and antioxidants can resist the harm caused by oxygen free radicals (Zhang J. et al., 2019; Forman and Zhang, 2021). In recent years, many traditional Chinese medicines and their active components have been reported to improve the activity of antioxidant defense enzymes, reduce or eliminate the generation of oxygen-derived free radicals, and exert antioxidant effects, which have been widely used in healthcare and disease prevention (Xu et al., 2019).

1,1-Diphenyl-2-picrylhydrazyl (DPPH), superoxide anion radicals and hydroxyl radicals are widely used to evaluate the free radical scavenging ability of natural compounds (Chen et al., 2014). SMP have been proved to have significant scavenging effects on a variety of free radicals, such as hydroxyl radicals, superoxide anions radicals and hydrogen peroxide. SMP extracted by RSM with ultrasound has significant scanning the DPPH to exert antioxidant activity (Jiang et al., 2014). The IC50 of SMP on DPPH and hydroxyl free radical scavenging ability were 0.991 mg/mL and 4.007 mg/mL, respectively. It could regulate the activity of antioxidant enzymes *in vivo* and had good antioxidant activity (Jing et al., 2022). Overproduction of reactive oxygen species (ROS) and oxidative stress play key roles in cerebral ischemia reperfusion (I/R) injury. In a rat cerebral I/R injury experiment, SMP reduced the production of ROS, increased the activities of superoxide dismutase (SOD), catalase (CAT) and glutathione peroxidase (GSH-Px), reduced the production of malondialdehyde (MDA), and played a protective role against brain I/R injury due to its antioxidant effect (Tu et al., 2013). Three polysaccharides (SMP-1, SMP-2 and SMP-4) were extracted from *Salvia miltiorrhiza* by hot water method, ultrasonic method and enzymatic method, which showed significant antioxidant activity (Wu et al., 2012). However, SMP-3 extracted by alkali method showed weak antioxidant activity, indicating that different extraction methods would affect biological activity. The above studies indicated that SMP may be a potential natural antioxidant. However, there is a large gap between clinical application and basic research, which needs to be further explored.

### 4.2 Hepatoprotective effect

The carbon tetrachloride (CCl4)-induced liver injury model is often used to evaluate the liver protective effect of SMP *in vivo* and *in vitro*. Han et al. (2019) established a chicken model of acute liver injury *in vivo* by using 2 mL/kg 50% CCl4. Treatment with SMP increased the levels of albumin, total protein and glutathione (GSH) and reduced the levels of alanine aminotransferase (ALT), aspartate aminotransferase (AST) and MDA. *In an in vitro* study, the hepatocyte injury model was established by adding CCl4 into hepatocytes. After treatment with SMP, the contents of ALT, AST and MDA were decreased, and the levels of GSH and cytochrome P450 (CYP450) were upregulated. The pathological process of liver injury includes not only the direct effect of viruses but also the disturbance of the immune system. When the concentration of SMP is 90–360 mg/kg, it has a certain protective effect on immune liver injury in mice, mainly by reducing the levels of ALT, AST and nitric oxide (NO) and improving tumor necrosis factor (TNF-α) and interleukin-1β (IL-1β) to reduce the degree of liver injury (Song et al., 2008). It is also confirmed that SMP alleviate the immunological injury in the liver by down-regulating the production of nuclear factor-kappa B (NF-κB), inducible nitric oxide synthase (iNOS) and MDA, and up-regulating the enzymes of the citric acid cycle (Sun et al., 2011).

Flufenicol (FFC) is often used to prevent and reduce mortality in chickens. However, studies have found that FFC could promote oxidative stress and accelerate hepatocyte apoptosis, thus affecting the liver function of chickens (Han et al., 2020; Liu et al., 2022). In a model of liver injury caused by FFC in broilers, SMP alleviates oxidative stress and inflammatory injury in the liver, improves liver function and plays a liver protective role, which provides theoretical support for the study of SMP on the treatment of drug-induced liver injury (Geng et al., 2022). Endotoxin metabolism activates the Toll-like receptor 4 (TLR4) pathway in the liver, leading to the increased expression of inflammatory cytokines and chemokines and then to liver inflammation. In a mouse liver injury model induced by lipopolysaccharide (LPS)/galactoamine (d-GalN), SMP alleviated liver inflammation by inhibiting the TLR4/MyD88 inflammatory signaling pathway (Wang X. et al., 2019). Non-alcoholic fatty liver disease (NAFLD) is kind of chronic liver disease, and the incidence rate has been increasing. At present, there is no effective drug treatment for NAFLD. SMP improves the progression of NAFLD by improving intestinal function and reducing serum LPS through the gut-liver axis (Li et al., 2022). In addition, SMP combined with probiotics improves NAFLD by regulating intestinal flora and improving insulin resistance (Wang et al., 2020).

### 4.3 Anti-tumor effect

Polysaccharides play an antitumor role by enhancing immunity, inhibiting tumor cell growth, inducing tumor cell apoptosis and preventing tumor cell migration and metastasis in the body (Moradali et al., 2007). A large number of studies have shown that SMPs have significant anti-tumor activities *in vivo* and *in vitro*.

SMP has a concentration-dependent inhibitory effect on human colorectal cancer LoVo cells by blocking cell cycle in S phase and inducing apoptosis through ROS-dependent pathway (Wang et al., 2018). Meanwhile, SMP have significant anti-proliferative activity against the human breast cancer cell line Bcap-37 and the human esophageal cancer cell line Eca-109, especially at a concentration of 0.30 mg/mL (Jiang et al., 2014). The immune response ability of the body is closely related to the occurrence of malignant tumors and plays an irreplaceable role (Zamarron and Chen, 2011). a polysaccharide (SMP-W1) from *Salvia miltiorrhiza* inhibited the proliferation of hepatocellular carcinoma H22 cells, especially at a high concentration of 400 μg/mL. Moreover, SMP-W1 significantly inhibited tumor growth in mice, increased the activity of antioxidant enzymes in mice, and improved the body weight, spleen index and thymus index of tumor mice to exert an anti-tumor effect, these results suggest that SMP-W1 may be an antitumor drug with immunomodulatory ability (Liu et al., 2013). A polysaccharide (SMPA) extracted from the root of *Salvia miltiorrhiza* in the rat model of gastric cancer significantly stimulates the proliferation of splenocytes, enhances the activity of natural killer (NK) cells and cytotoxic T lymphocytes (CTL), and improve the phagocytic ability of macrophages, thus playing an anticancer role (Wang et al., 2014).

### 4.4 Cardioprotective effect

*Salvia miltiorrhiza* has been used as a Chinese herbal medicine in the treatment of cardiovascular disease for thousands of years. Traditional Chinese medicine holds that *Salvia miltiorrhiza* has the effect of promoting blood circulation and removing blood stasis (Chen F. et al., 2017; Wang et al., 2017). SMP, one of the major active ingredients of *Salvia miltiorrhiza*, has been found to have a protective effect on cardiovascular diseases. In a rat model of myocardial I/R injury induced by occlusion of the left anterior descending coronary artery for 30 min and reperfusion for 4 h, oral administration of 400 mg/kg and 800 mg/kg SMP reduced serum lactate dehydrogenase (LDH), creatine kinase (CK) and MDA levels, improved myocardial SOD, Na + -k + -ATPase and Ca + -Mg + -ATPase activities and inhibited cardiomyocyte apoptosis, These results indicated that SMP had a protective effect on myocardial I/R injury in rats by improving oxidative stress and inhibiting myocardial apoptosis (Song et al., 2013). Oxidative stress in cardiomyocytes is closely related to the occurrence of cardiovascular disease. *In vitro* experiments found that SMP could inhibit mitochondrial dysfunction, activate caspase protein expression, and enhance antioxidant capacity, which provides a theoretical basis for further study of SMP in the treatment of cardiovascular diseases (Geng et al., 2015a). To further clarify the cardioprotective effect of SMP, in the isoproterenol (ISO)-induced myocardial infarction rat model, pretreatment with SMP (100 and 400 mg/kg) significantly increased serum CK, creatine phosphokinase MB (CK-MB), total cholesterol, triglyceride and low density lipoprotein cholesterol (LDL-C) levels, and enhanced SOD, CAT and GSH-Px activities, which showed that oral administration of SMP can play a protective role against ISO-induced heart injury in rats through enhancing endogenous antioxidant and anti-hyperlipidemia activity (Geng et al., 2015b).

### 4.5 Anti-inflammatory effect

Inflammation is the immune defense response of the body to pathogens and tissue damage, which is mainly produced by stimulating immune cells at the injury site to release inflammatory mediators (Roe, 2021). Many plant polysaccharides, such as *Momordica charantia* polysaccharide and *Laminaria japonica* polysaccharides, have shown good anti-inflammatory activity (Deng et al., 2014; Peng et al., 2015). Endotoxin (LPS), the main component of the cell wall of Gram-negative bacteria, can induce macrophages to differentiate into M1 macrophages, promote the release of inflammatory cytokines, and cause inflammation and tissue damage. SMP could significantly inhibit the mRNA expression of TNF-α, interleukin-6 (IL-6), iNOS and cyclooxygenase (COX)-2 and the protein expression of NF-κB, p-p65 and p-IkBa in RAW264.7 macrophages induced by LPS (Han et al., 2018). *Staphylococcus aureus* is an important pathogen that causes mastitis in dairy cows. When studying the effectiveness of SMP against mastitis, it was found that SMP supplementation could inhibit the activities of myeloperoxidase (MPO) and N-acetyl-β-D-glucosaminidase (NAGase) and reduce the expression of IL-6, IL-1β and TNF-α inflammatory factors in rats with mastitis. and protect rat mammary gland tissue from excessive inflammatory reaction damage (Zhang et al., 2022). In addition, SMP could increase the level of anti-inflammatory cytokines (IL-2, IL-4 and IL-10) and inhibit the expression of proinflammatory cytokines (IL-6 and TNF-α) to play an antitumor role (Wang et al., 2014).

### 4.6 Immunoregulatory effect

Immune regulation is the body’s immune system to recognize self and foreign substances, and through the immune system to remove antigen foreign body immune response to maintain the physiological balance of the body. The occurrence and development of many diseases (such as infection, inflammation, cancer) are closely related to the abnormal immune system (Yu et al., 2018). In recent years, many studies have found that SMP exerts its immunomodulatory activity by regulating the activity of cytokines, activating immune organs, and stimulating the expression of lymphocytes and macrophages. Cyclophosphamide can inhibit the immune system, and SMP could effectively enhance the activity of immune cells in mice treated with cyclophosphamide and increase the proportion of lymphocyte subsets and the expression level of immune-related cytokines in a dose-dependent manner (Ji et al., 2021). Humoral immunity is an important specific immune response mediated by B cells and is one of the main factors for the body to resist infectious diseases. Immunoglobulin G (IgG), immunoglobulin M (IgM) and immunoglobulin A (IgA) are the main immunoglobulins produced by activated B lymphocytes, reflecting the humoral immune status (Khalifeh et al., 2009). FFC combined with SMP could significantly increase the levels of IgG, IgM and IgA in chicken serum, increase the number of leukocyte subtypes in blood, and enhance the immune response of chickens (Han et al., 2021). Equally, Jiang et al. also found that adding 1.5 g/kg SMP to weaned piglets improved immune ability by increase the content of IgG, IgM and IgA (Jiang et al., 2020). *In vitro*, SMP could increase the activity of macrophages and lymphocytes, increase the concentration of lysozyme and nitric oxide (NO) in serum, and then enhance the immune function and disease resistance of sturgeon (Chen et al., 2022). In addition, SMP significantly improved the body weight, spleen index and thymus index of tumor-bearing mice to improve the immune response (Liu et al., 2013). These studies provide evidence for the use of SMP as a natural immune stimulant. However, current studies on the immune activity of SMP are still limited to the animal and cell levels, and further studies are needed to determine whether it can be used in clinic.

### 4.7 Other significant effects

Zhang et al. (2012) studied the mechanism of SMP in preventing and treating T2DM in the insulin resistance (IR) rat model. SMP increased the expression of CAT, SOD, and GSH-Px, improved the insulin sensitivity index, and attenuated the morphological injury of the liver and pancreas to protect against the development of T2DM and improve IR. Shen et al. (2015) studied the effect of SMP on boar sperm motility and found that frozen semen containing 0.4 mg/mL SMP could protect boar sperm from oxidative damage and improve sperm motility and litter size in artificial insemination of sows. As a novel broad-spectrum antibiotic for chloramphenicol in livestock, FFC could not only cause liver function damage but also lead to abnormal renal function, which may be caused by oxidative stress and abnormal renal cell apoptosis (Wang et al., 2021a; Wang et al., 2021b). Multiple studies have found that SMP has a protective effect on the kidney by reducing oxidative stress in the kidney, promoting drug metabolism enzymes and inhibiting apoptosis of renal tissue (Lu et al., 2022; Wang et al., 2022) Adding 1.5 g/kg SMP to weaned piglets significantly increased the ratio of ileum villus height to crypt depth, and changed the density and population of intestinal microflora, which improved the digestion and absorption ability of weaned piglets (Jiang et al., 2020).

## 5 Conclusion and future perspectives

As the main active ingredient of *Salvia miltiorrhiza*, SMPs have attracted increasing attention and research in recent years due to its extensive pharmacological action and biological activity. Based on previous studies, it was found that SMPs are a kind of water-soluble heteropolysaccharide with a molecular weight between 2 and 1300 kDa. Hot water extraction is the most commonly used extraction method because of the high hydrophilicity of SMPs. In addition, alkaline extraction, ultrasonic extraction, enzymatic extraction and microwave extraction are also used to extract SMPs. The SMPs extracted by existing methods contains protein, pigment and other impurities, which need to be further removed by filtration, dialysis, deproteinization and other methods. Different sources and different methods of extraction and purification of *Salvia miltiorrhiza* lead to different structural characteristics and pharmacological activities of the extracted SMPs. Therefore, the extraction and purification methods of SMPs need to be further studied.

The structural characteristics of polysaccharides determine their biological activities. The diversity of monosaccharide composition, glycosidic bond type and connection mode leads to the complexity and diversity of polysaccharide structure, which further leads to the extensive biological activity of polysaccharides. However, there are few studies on the relationship between biological activity and structure of SMPs, which may limit the development and utilization of SMP. further study on the relationship between SMPs structure and biological activity could provide theoretical support for further development and utilization of SMPs.

SMPs have a wide range of biological activities, such as immune regulation, antioxidation, kidney protection, anti-inflammation, heart protection, anti-tumor, anti-diabetes and liver protection. Different pharmacological actions interact. For example, the protective effect of the liver is realized by anti-inflammation and antioxidation, and immune regulation could increase the anti-tumor properties of SMPs. However, the research on SMPs are mainly limited to *in vitro* and *in vivo* experiments at present, and there are few reports in humans. Therefore, further studies on the efficacy and safety of SMPs in humans are necessary.

In this paper, the extraction, purification, structure and pharmacological properties of SMPs are reviewed, and points out its great value in medicine and healthcare products. With more comprehensive and in-depth research on SMPs, it is believed that SMPs will have a broad prospect and application market in biomedical and food applications.
